# Enhanced peripheral tissue oxygenation and hemoglobin concentration after a high-fat meal measured with spatial frequency domain imaging

**DOI:** 10.1117/1.BIOS.1.2.025004

**Published:** 2024-09-12

**Authors:** Anahita Pilvar, Jorge Plutzky, Darren Roblyer

**Affiliations:** a Boston University, Department of Electrical and Computer Engineering, Boston, Massachusetts, United States; b Brigham and Women’s Hospital and Harvard Medical School, Department of Medicine, Boston, Massachusetts, United States; c Boston University, Department of Biomedical Engineering, Boston, Massachusetts, United States

**Keywords:** cardiovascular disease, diffuse optics, nutrition, spatial frequency domain imaging (SFDI), triglycerides

## Abstract

**Significance:**

The magnitude and temporal dynamics of changes in blood nutrient and lipid levels following a high-fat meal have been previously shown to be an important indicator of current and future cardiovascular health and disease. Measurement of circulating nutrients and lipids currently requires invasive blood draws. The development of a non-invasive method for continuous monitoring of postprandial (i.e., after-meal) changes may assist in enhancing cardiovascular health management, dietary monitoring, and identification of disease-promoting factors. Spatial frequency domain imaging (SFDI) is a non-contact, label-free optical technique that can quantify tissue optical properties and hemodynamics in vivo. We hypothesized that SFDI may track the postprandial state in peripheral tissue.

**Aim:**

We aim to investigate the relationship between postprandial factors, namely triglycerides and glucose, and the optical properties and oxygenation of peripheral tissue measured with SFDI.

**Approach:**

Fifteen healthy volunteers consumed both a low- (2 g) and high- (60 g) fat meal on different days. A custom SFDI device was used to measure the dorsal hand surface of volunteers before the meal and each hour for 5 h after the meal. Measurements were taken at 730, 880, and 1100 nm. Longitudinal postprandial changes in tissue optical properties were correlated with changes in blood triglycerides and glucose levels as well as blood pressure, heart rate, and room temperature. A machine-learning model was trained to estimate triglyceride levels from SFDI metrics.

**Results:**

Several SFDI metrics increased and peaked 3 to 4 h following the high-fat meal, including tissue oxygen saturation (${\mathrm{StO}}_{2}$) and oxyhemoglobin (${\mathrm{HbO}}_{2}$) concentration, and were substantially different from the low-fat cohort (p<0.05 at 3 h). The increases were large, >5% for ${\mathrm{StO}}_{2}$ and >10% for ${\mathrm{HbO}}_{2}$ concentration on average. The temporal changes in these metrics broadly tracked triglyceride levels, which peaked at 3 h post-meal. The predictive model accurately estimated blood triglyceride levels (RMSE 40  mg/dL).

**Conclusion:**

These findings suggest that SFDI could serve as a powerful non-invasive tool to monitor postprandial hemodynamics. In the future, SFDI measurements may help enhance cardiovascular disease prediction and management.

Statement of Discovery This work utilizes spatial frequency domain imaging to demonstrate for the first time that peripheral tissue oxygen saturation and hemoglobin concentrations increase after a high fat meal. This could lead to a new non-invasive optical method to track diet-induced changes in cardiovascular physiology.

## 1 Introduction

Meal consumption induces a variety of acute physiological changes within the body, and the several-hour period immediately following a meal, known as the postprandial state, plays a crucial role in determining cardiovascular health.^1^ As such, measuring dynamic responses to meal intake affords a unique and distinct opportunity for assessing cardio-metabolic risk. One of the most prominent effects of meal consumption is the alteration in plasma levels of lipids and nutrients. For example, blood plasma concentrations of glucose, triglycerides, amino acids, dietary cholesterol, and electrolytes typically increase within hours in the postprandial period, typically considered within 4 h of eating. Each of these circulating factors has unique temporal postprandial dynamics in terms of their concentration levels in the blood. For example, while glucose levels usually spike in less than 2 h,^2^ triglyceride levels often stay elevated longer, typically peaking at 3 to 5 h.^3^ Notably, these dynamic changes are also altered in various disease as well as pre-disease states. For example, individuals with diabetes typically demonstrate abnormal glucose responses to a meal challenge, which is used clinically to diagnose diabetes and can also be observed in the pre-diabetic state.^4^ Abnormal postprandial elevations in plasma triglycerides after a high-fat meal are a strong indicator of an increased risk of developing cardiovascular disease (CVD).^5^^,^^6^ Studies have suggested that the dynamic changes in triglyceride levels over several hours post-meal are more indicative of cardiovascular risk than a single fasting measurement.^5^^,^^6^ A major practical limitation in obtaining postprandial measurements is the requirement of serial blood draws followed by laboratory-based chemical assays, as manifested through cost, laboratory time, patient pain, and inconvenience, all being factors that have limited the use and development of postprandial measurement. The development of a non-invasive method to detect and monitor relevant postprandial changes could enhance screening accessibility for at-risk individuals and provide critical insights for individuals aiming to optimize their dietary choices and manage their cardiovascular health.

Limited prior non-invasive postprandial measurement techniques have been reported. One common method for assessing peripheral vascular changes is through ultrasound-based flow-mediated dilation studies, typically conducted within the first 2 h after a meal.^7^^–^^10^ These studies measure the dilation of major conduit arteries in the arm such as the brachial artery following the release of a blood pressure cuff. These studies have revealed that consumption of a high-fat meal is associated with a reduction in large-vessel endothelial function. These methods typically require a repeated cuff-based measurement and an experienced ultrasound operator, limiting their use as part of the standard of care. Other studies have shown alterations in core and peripheral blood flow following a meal, especially in the first 1 to 2 h after the meal.^11^^,^^12^ Little is currently known about how peripheral tissue optical properties and hemodynamics change in the postprandial window, which can last as long as 5 to 6 h.

In this work, we investigate the utility of a non-invasive optical measurement technique known as spatial frequency domain imaging (SFDI) to observe postprandial alterations in the optical properties of peripheral tissue for the first time. The non-contact nature of SFDI enables straightforward subject measurements over a widefield of tissue. Here, we describe the results of a human study in which healthy volunteers were given both low- and high-fat meals and monitored during the postprandial state every hour for 5 h with SFDI. We correlate changes in SFDI-derived metrics to blood nutrient and lipid concentrations and other hemodynamic measures such as heart rate and blood pressure. Our findings offer valuable insights into the noninvasive measurement of meal-induced changes in tissue optical properties and present a potential noninvasive tool for dietary management in both healthy individuals and those with CVDs.

## 2 Methods

### 2.1 Subject Eligibility and Enrollment

The study was conducted in compliance with an institutionally approved review board protocol at Boston University (protocol number 4698). Informed consent was obtained from all participants prior to the study. Fifteen healthy subjects (eight males and seven females, age 27±4) were recruited. Each subject participated twice in the study, once after a low-fat meal and once after a high-fat meal. The order in which subjects participated in high-fat or low-fat meal study was randomized. Subjects who were <18 years old or who had a prior history of hypotension (low blood pressure), low blood sugar, dizziness, fainting, and type 1 or 2 diabetes were excluded from the study. Participants were asked to fill out a questionnaire containing age, weight, height, race and ethnicity, and skin tone information prior to the start of the study.

### 2.2 Postprandial Experiment Procedure

**Fig. 1:**
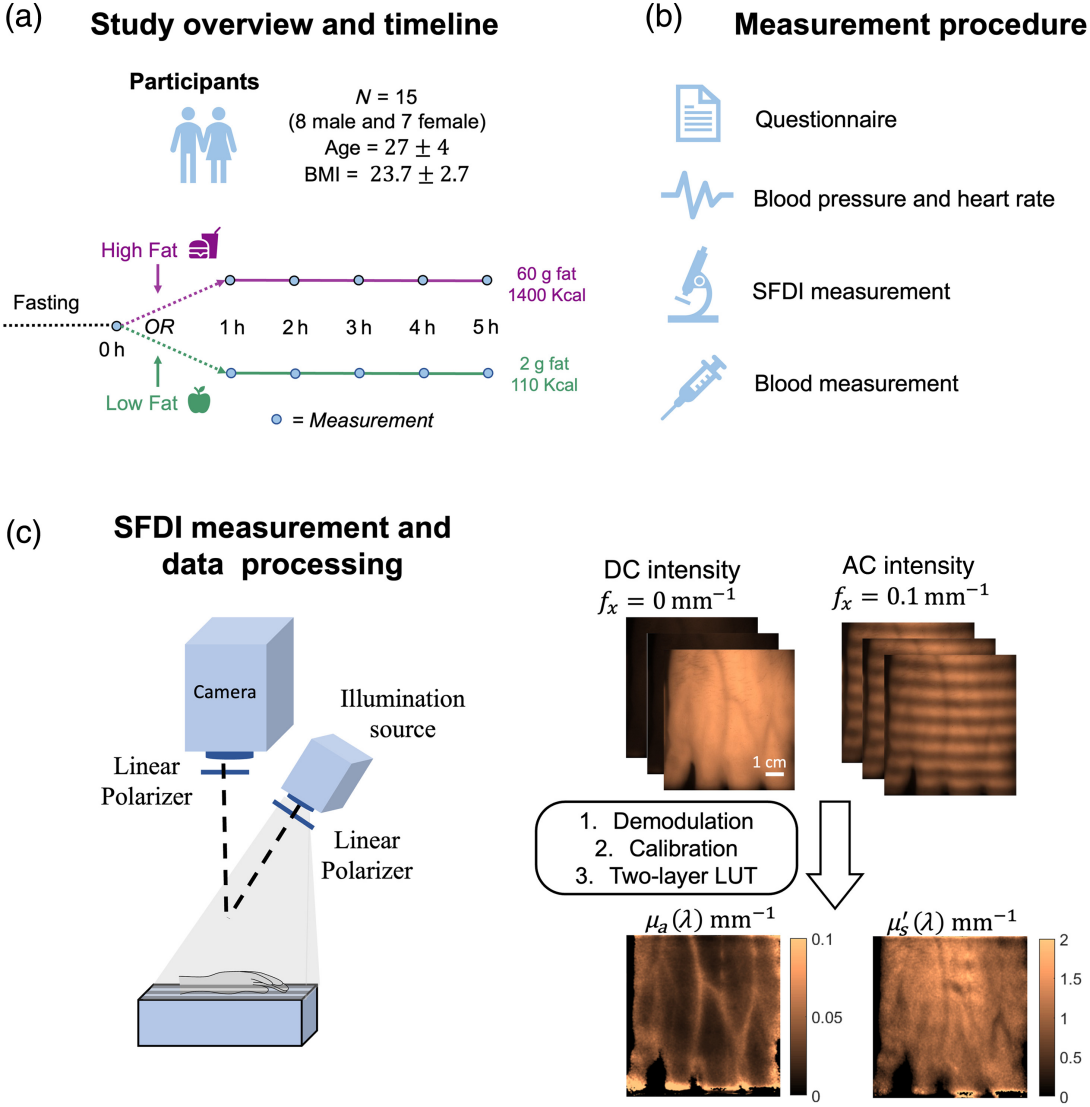
(a) Study timeline. (b) Measurement procedure. (c) Schematic diagram of three-LED SFDI system, SFDI data acquisition, and processing flowchart. The top row shows the raw images captured at the spatial frequency of DC and $0.1\;{\mathrm{mm}}^{-1}$ from the dorsal surface of a subject’s hand at 880 nm. The captured images are demodulated and calibrated, and the diffuse reflectance data is converted into absorption and reduced scattering coefficients using a lookup table. The bottom row shows the $\mu_{a}$ and $\mu_{s}^{\prime }$ maps.

The study procedure is shown in Fig. 1. Subjects who agreed to participate in the study were asked to complete 10 h of overnight fasting. For the high-fat meal study, subjects were provided a meal the next morning that contained ∼60  g of fat and 1400 KCal. Subjects had the option to choose between breakfast sandwiches from Dunkin Donuts (bacon egg and cheese or sausage egg and cheese on croissant) or a big breakfast option from McDonald’s combined with two protein shakes. For the low-fat meal study, subjects were provided oatmeal (2 g of fat, 110 KCal) in the morning of the study. The nutrient content of the meals is shown in Table S1 in the Supplementary Material. Subjects were monitored following the measurement procedure at the baseline (before consuming the meal in the morning) and every hour for 5 h after the meal.

During each SFDI measurement, participants were instructed to sit in a chair and place their right hand into a silicone hand holder positioned under the SFDI system. SFDI measurements were conducted on the dorsal surface of the participants’ hands while they were asked to keep their hands still in the holder. The dorsal hand surface was selected for SFDI measurements due to the visibility of superficial blood vessels. In addition, this area offers convenience for repeated measurements and minimal discomfort for subjects. SFDI patterns were projected at spatial frequency pair of DC and $0.1\;{\mathrm{mm}}^{-1}$ at three wavelengths of 730, 880, and 1100 nm. Glucose and lipid profiles were then assessed using an in-lab lipid analyzer (Alere Cholestech LDX Analyzer, Waltham, Massachusetts, United States). A fingerstick procedure was performed on the participant’s finger to collect several small drops of blood using a lancet within 10 s. The blood sample was then placed in a cassette and inserted into the Alere system to obtain the full blood profile and glucose concentration.

After finishing the procedure in the fasting state, the subjects were asked to eat either a low-fat (control group) or a high-fat meal (known to induce increased blood lipids). The measurement procedure was then repeated every 1 h for a total of 5 h after the meal. In addition, blood pressure, heart rate, and room temperature were recorded for a subset of subjects (10 high fat and 10 low fat) at every time point. Blood pressure and heart rate were measured using an automated cuff (BPM Connect, Withings). Subjects were free to leave the lab between measurements. Participants were told not to eat for the duration of the study, but they were allowed to drink water. Room temperature data were not recorded for subject 12 in the low-fat meal category, and blood profile data for subject 8 in the same category were missing at the 4-h post-meal timepoint due to complications with the fingerstick blood collection procedure.

### 2.3 SFDI Acquisition and Data Analysis

Details regarding the SFDI system and data processing have been described elsewhere.^13^ Briefly, SFDI is a label-free non-contact diffuse optical imaging modality that provides measurements of optical properties (absorption and reduced scattering coefficients) of a biological sample from a large field of view on a pixel-by-pixel basis. In this study, a custom-built LED-based SFDI system was used to perform all the SFDI measurements.^14^ The system utilizes two LEDs in the near-infrared wavelength band (730 and 880 nm) and one LED in the shortwave infrared wavelength band (1100 nm) as the illumination source. Both planar and sinusoidal spatial patterns of light at $0.1\;{\mathrm{mm}}^{-1}$ were projected on a sample using a digital micro-mirror device (DMD), and the remitted light was captured by a germanium CMOS camera (TriWave, Infrared Laboratories, Inc., Peabody, Massachusetts, United States). A three-phase illumination and demodulation technique was used to extract the tissue response at the given spatial frequencies. The field of view (FOV) was 7×11  cm. The exposure time for all three wavelengths was set to 100.925 ms.

Intralipid (Baxter, Deerfield, Illinois, United States) with 10% lipid concentration was used as a calibration phantom with known optical properties to remove the instrument response. The scattering properties of intralipid at a 10% lipid concentration were adapted from literature,^15^ and the absorption properties were calculated using Beer’s law for the wavelengths of interest.^16^^,^^17^ Optical absorption ($\mu_{a}$) and reduced scattering coefficients ($\mu_{s}^{\prime }$) were estimated at each illumination wavelength from a lookup table generated by Monte Carlo simulations with calibrated diffuse reflectance maps at two spatial frequencies as inputs. In addition, the extracted $\mu_{a}$ values at all three wavelengths were used to extract hemoglobin information, including the concentration of oxyhemoglobin (${\mathrm{HbO}}_{2}$) deoxyhemoglobin (Hb), total hemoglobin (THb), and hemoglobin oxygen saturation (${\mathrm{StO}}_{2}$) using Beer’s law. A 2  cm×2  cm ROI was selected in the center of the hand and the average $\mu_{a}$ and $\mu_{s}^{\prime }$ at the three measurement wavelengths, and the concentrations of ${\mathrm{HbO}}_{2}$, Hb, THb, ${\mathrm{StO}}_{2}$, and the a and b scattering parameters were calculated inside the ROI at each time point. The a and b parameters were determined from a power law fit to the three measured $\mu_{s}^{\prime }$ values. In addition, vascular and microvascular areas were segmented using MATLAB fibermetric function and thresholding technique to compare the changes that happen over different anatomic regions.^14^^,^^18^ We specifically assessed the vascular size of large superficial vessels at each timepoint post-meal, applying a consistent threshold value for segmentation across all timepoints. We also examined the full width at half maximum (FWHM) across prominent superficial vessels to observe any postprandial alterations. A more detailed description of the segmentation process and FWHM calculation is described in the Supplementary Material.

In addition to the absolute optical properties and hemoglobin values, a variety of composite metrics of optical properties were also calculated. All SFDI features were normalized to the value at the baseline to compare the relative changes over time as well as comparing the two categories (low fat versus high fat). Data exhibiting motion artifacts were excluded from the processing analysis to ensure the integrity and accuracy of the results; eight out of 180 (4.44%) optical measurement sessions have excluded datapoints for this reason. The exclusion of the baseline measurement for subject 12, due to motion artifacts after a high-fat meal, necessitated the removal of all timepoints for subject 12 as longitudinal data were normalized to the baseline measurement. Consequently, to maintain the matched low-fat and high-fat dataset, we also excluded the low-fat meal data for subject 12. All data were processed using MATLAB (R2021b, The Mathworks Inc., Natick, Massachusetts, United States) or Python.

### 2.4 Statistical Analysis

Descriptive data are presented as mean ± standard error (SE). Statistical analysis was conducted using the “anovan” function in MATLAB to compare the SFDI-derived parameters between two groups (high-fat meal and low-fat meal) over six timepoints and between timepoints at each group. The analysis examined the interaction effect between the group and time factors. Post hoc analysis was performed using the “multcompare” function to determine pairwise differences between the groups at each time point. A p-value<0.05 was considered statistically significant.

### 2.5 Prediction Model

A prediction model was trained to output estimated triglyceride concentrations in the postprandial state using SFDI metrics as inputs. To achieve this, 16 normalized SFDI metrics were used as inputs and trained against triglyceride concentrations relative to their baseline values. A five-fold cross-validation approach was utilized in which the dataset was divided into five equal parts. During each iteration, we trained an XGBoost model on the training dataset and then used it to predict the normalized triglyceride concentrations on the test data. These predictions were then scaled to the original mg/dl scale using their corresponding baseline triglyceride concentrations. This process was repeated five times to ensure the robustness and reliability of our model. We then evaluated our model based on the accuracy of the predictions.

## 3 Results

### 3.1 Postprandial Changes After Low-Fat and High-Fat Meals

**Fig. 2:**
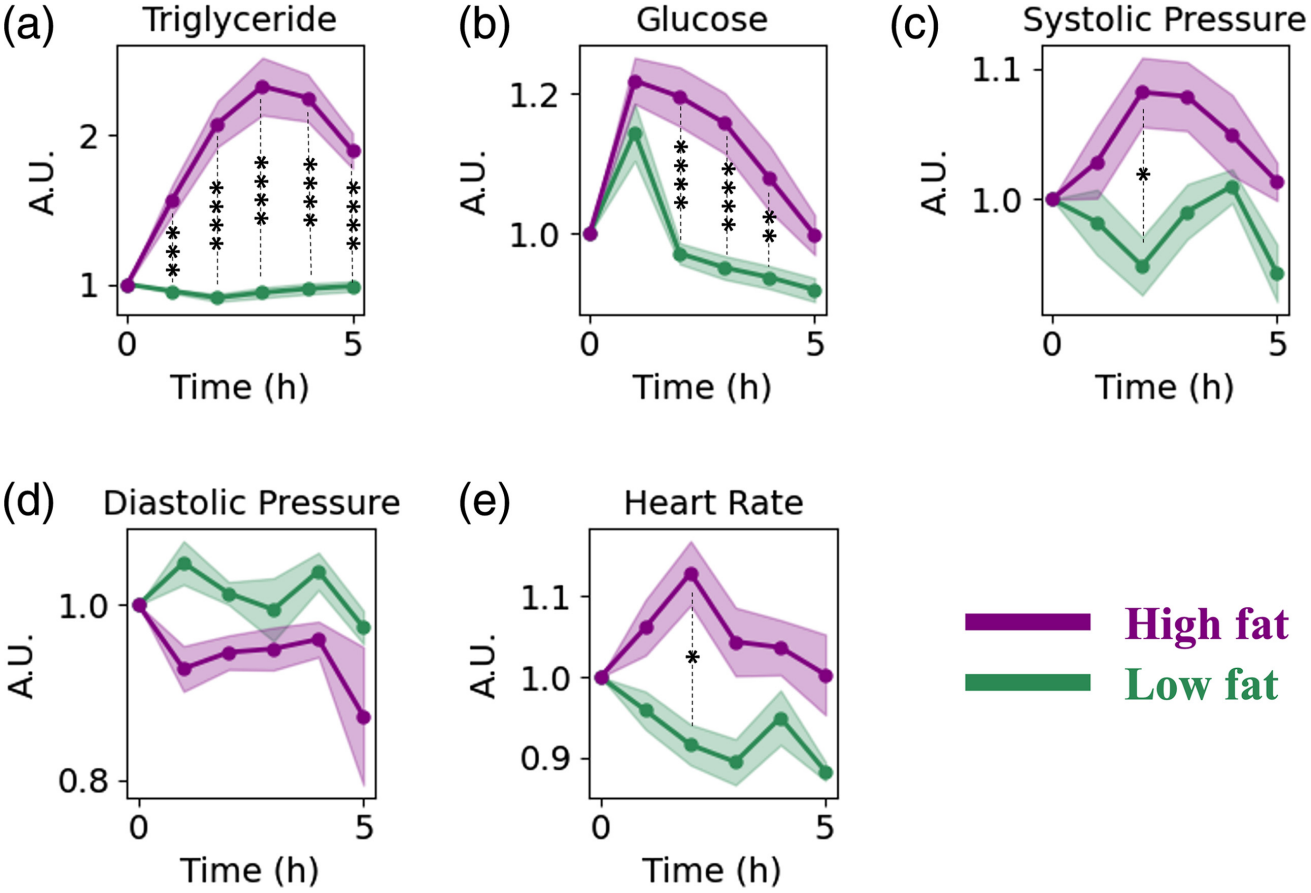
Relative changes in (a) triglyceride concentration, (b) glucose concentration, (c) systolic blood pressure, (d) diastolic blood pressure, and (e) heart rate compared with the fasting state (time point = 0) during the 5 h postprandial measurements after high-fat (purple) and low-fat (green) meal. Data shows the average values and the standard error as a shaded area. Asterisks show the level of significant differences between the two meal groups. p<0.0001 is shown with ****, p<0.001 is shown with ***, p<0.01 is shown with **, and p<0.05 is shown with *.

The effect of high-fat and low-fat meals on blood triglyceride, glucose, systolic pressure, diastolic pressure, and heart rate over time is shown in Fig. 2. All results are shown as relative changes from the baseline measurement of the specific parameter. A substantial increase in blood triglycerides occurred at all timepoints after the high-fat meal; by contrast, little change in triglycerides was evident after the low-fat meal across all timepoints. For the high-fat meal subjects, the triglyceride concentrations typically reached their peak 3 h after the meal consumption. These different trends led to statistical differences between high- and low-fat meal cohorts at every hour after the meal (p<0.001 for the first hour, p<0.0001 for 2 to 5 h). Glucose increased 1 h after the meal for both groups, with significant differences between the groups observed at hours 2 to 4 (p<0.01). No significant changes in blood cholesterol occurred following the meals. Systolic blood pressure increased after the high-fat meal, and a significant difference between high-fat and low-fat meals occurred 2 h after the meals (p<0.05). Diastolic blood pressure did not show any significant trend with time, but the heart rate increased after a high-fat and decreased after the low-fat meal with a significant difference between the two groups occurring 2 h after the meals (p<0.05). Within the cohort (high-fat or low-fat), changes from the baseline were not significant for either systolic or diastolic blood pressure or heart rate. Room temperature was recorded as temperature may affect blood flow in superficial tissue. Room temperature was not significantly different between low- and high-fat cohorts at any time, although there was a significant difference in room temperature at 5 h compared with baseline within the low-fat meal cohort, in which the room temperature increased 2.7±2.0°F.

**Fig. 3:**
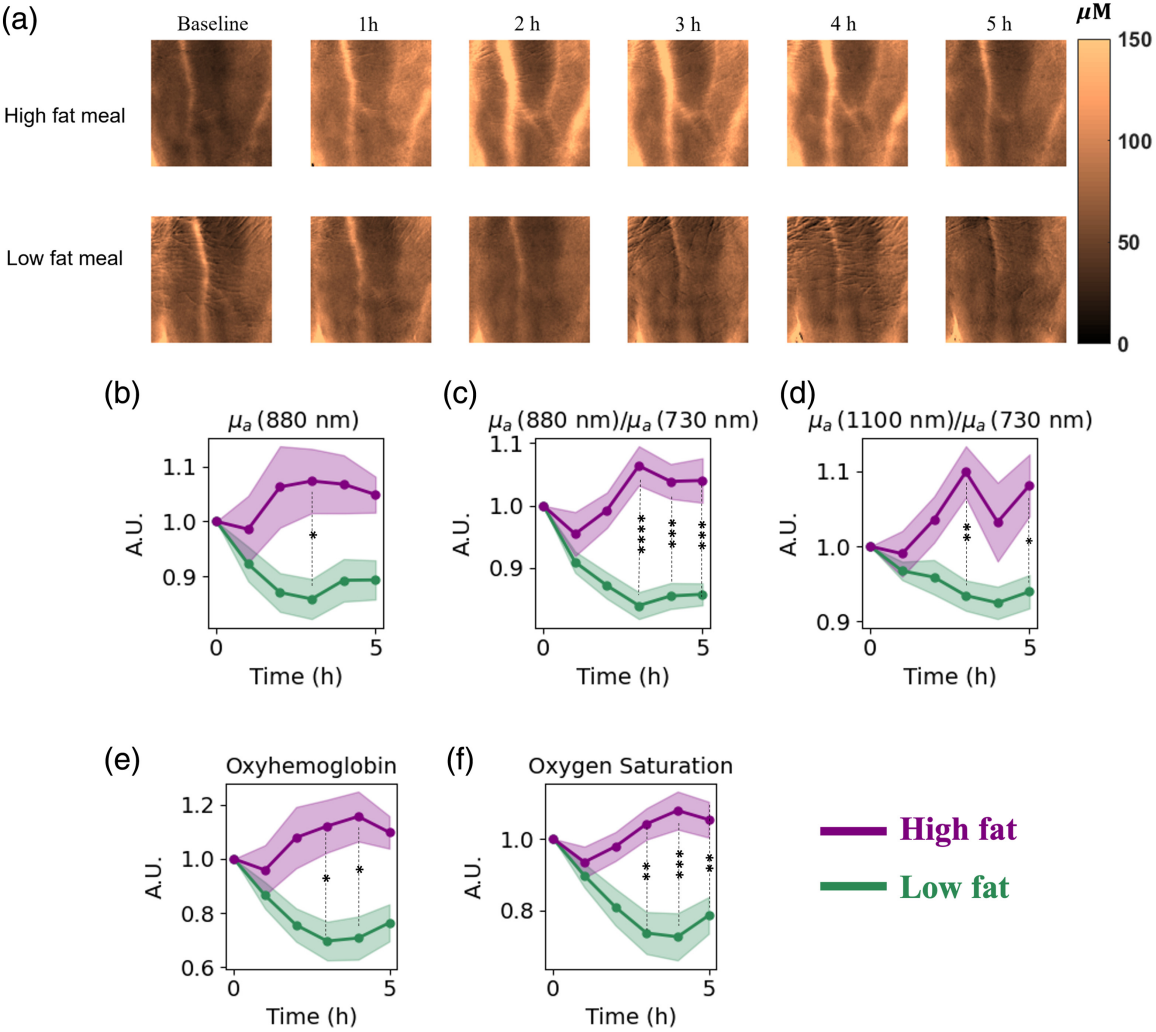
(a) ${\mathrm{HbO}}_{2}$ concentration maps at baseline and postprandial states for representative subjects after high-fat and low-fat meals showing an increase in ${\mathrm{HbO}}_{2}$ after a high-fat meal and a decrease in ${\mathrm{HbO}}_{2}$ after a low-fat meal. (b) Relative changes in $\mu_{a}$ at 880 nm, (c) $\frac{\mu_{a}(880\;\mathrm{nm})}{\mu_{a}(730\;\mathrm{nm})}$, (d) $\frac{\mu_{a}(1100\;\mathrm{nm})}{\mu_{a}(730\;\mathrm{nm})}$, (e) ${\mathrm{HbO}}_{2}$, and (f) ${\mathrm{StO}}_{2}$ compared with the fasting state (time point = 0) during the 5 h postprandial measurements after high-fat (purple) and low-fat (green) meals. Asterisks show the level of significant differences between the two meal groups. p<0.0001 is shown with ****, p<0.001 is shown with ***, p<0.01 is shown with **, and p<0.05 is shown with *.

The temporal changes and trends in SFDI-derived parameters over the 6-h experiment are shown in Fig. 3, including the ${\mathrm{HbO}}_{2}$ map of a representative subject at baseline and postprandial states after both meals [Fig. 3(a)]. The maps indicate an increase in ${\mathrm{HbO}}_{2}$ concentration in the vascular and microvascular regions of the subject after the high-fat meal. For this subject, using the 2  cm×2  cm ROI centered on the hand, the average ${\mathrm{HbO}}_{2}$ concentration increased from 43.2  μM at baseline to 94.6  μM at 3 h after the high-fat meal. Conversely, after the low-fat meal, there was a decline in ${\mathrm{HbO}}_{2}$ levels. The average ${\mathrm{HbO}}_{2}$ concentration changed from 54.8  μM at baseline to 51.5  μM 3 h after the low-fat meal.

The longitudinal relative changes of SFDI metrics that changed differentially after the low- and high-fat meals are shown in Figs. 3(b)–3(f). Interestingly, SFDI metrics peaked 3 or 4 h after the high-fat meal, which largely matches the timing when conventionally measured triglyceride concentrations also reached their peak (Fig. 2). In several cases, there were statistically significant differences in SFDI metrics between high-fat and low-fat meals at multiple timepoints after the meal.

A separate examination of large vessel and microvascular regions following segmentation showed no distinct variations in trends between these areas. In addition, a spatial analysis of vascular size and FWHM did not reveal any significant trends or differences in response to meals with varying fat content. The detailed FWHM results, alongside an illustrative segmentation map, are presented in Fig. S1 in the Supplementary Material.

**Fig. 4:**
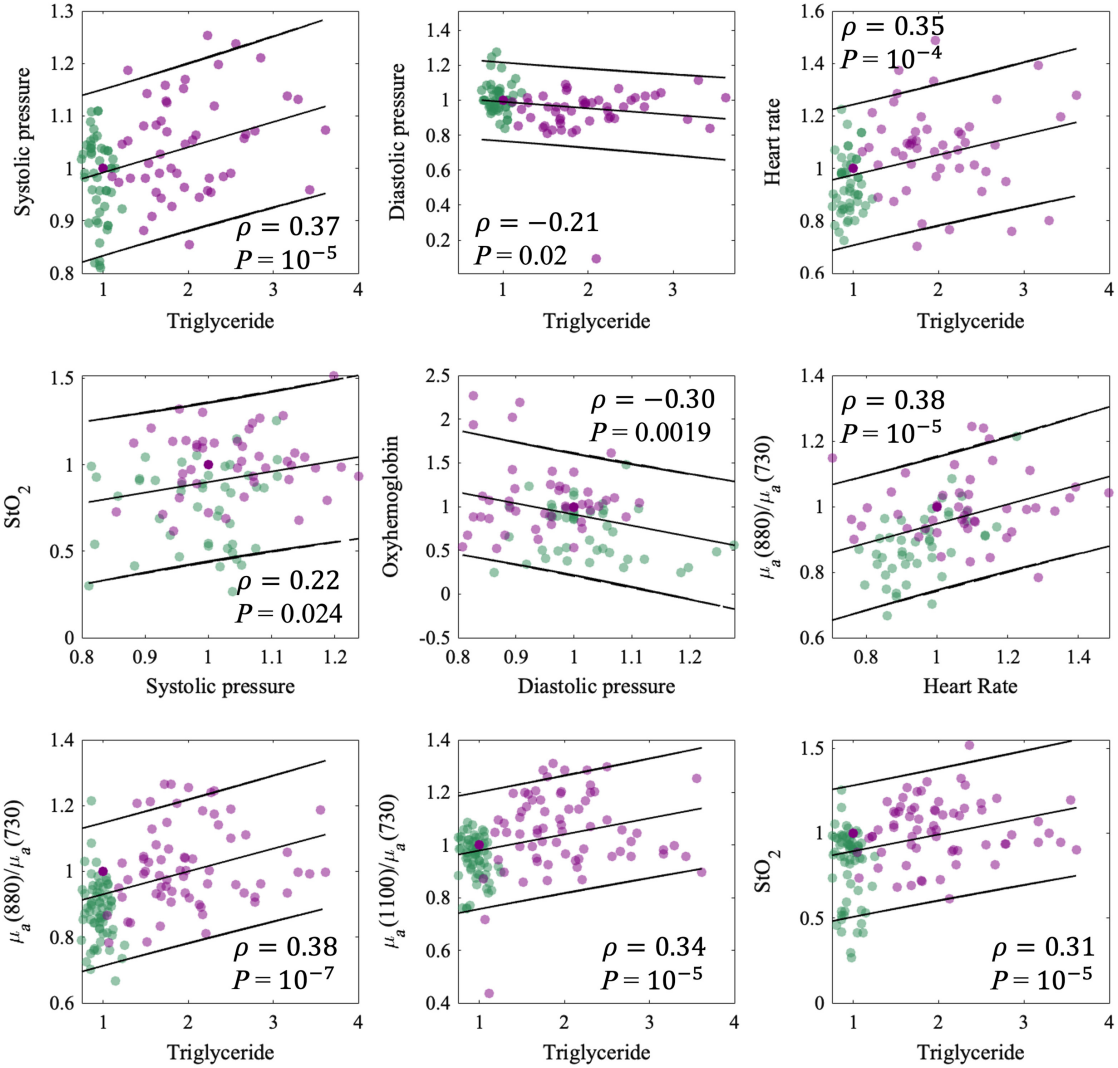
Correlation plots for highly correlated metrics combining all subjects from both high-fat and low-fat groups. All metrics were normalized to their respective baseline values. Black lines show the linear fit and 95% confidence interval. Green data points indicate measurements from the low-fat cohort, and purple from the high-fat cohort.

The notable correlations between blood triglyceride levels, blood pressure, and heart rate and SFDI-based metrics are shown in Fig. 4. The correlations for normalized parameters relative to baseline are provided. Overall, nine optical metrics measured with SFDI were correlated with blood triglyceride concentration with p-value<0.05. The highest correlations were found between triglycerides and $\frac{\mu_{a}(880\;\mathrm{nm})}{\mu_{a}(730\;\mathrm{nm})}$ as well as the heart rate and $\frac{\mu_{a}(880\;\mathrm{nm})}{\mu_{a}(730\;\mathrm{nm})}$, with a Pearson correlation coefficient = 0.38 in both cases, and $p\text{-}\text{value}=10^{-5}$ and $p\text{-}\text{value}=10^{-7}$. These results demonstrate that optical measurements measured with SFDI change in concert with triglycerides and hemodynamic parameters in the postprandial state. No scattering-based metrics correlated strongly with gold-standard measures.

### 3.2 Prediction of Blood Triglyceride Concentration from SFDI

**Fig. 5:**
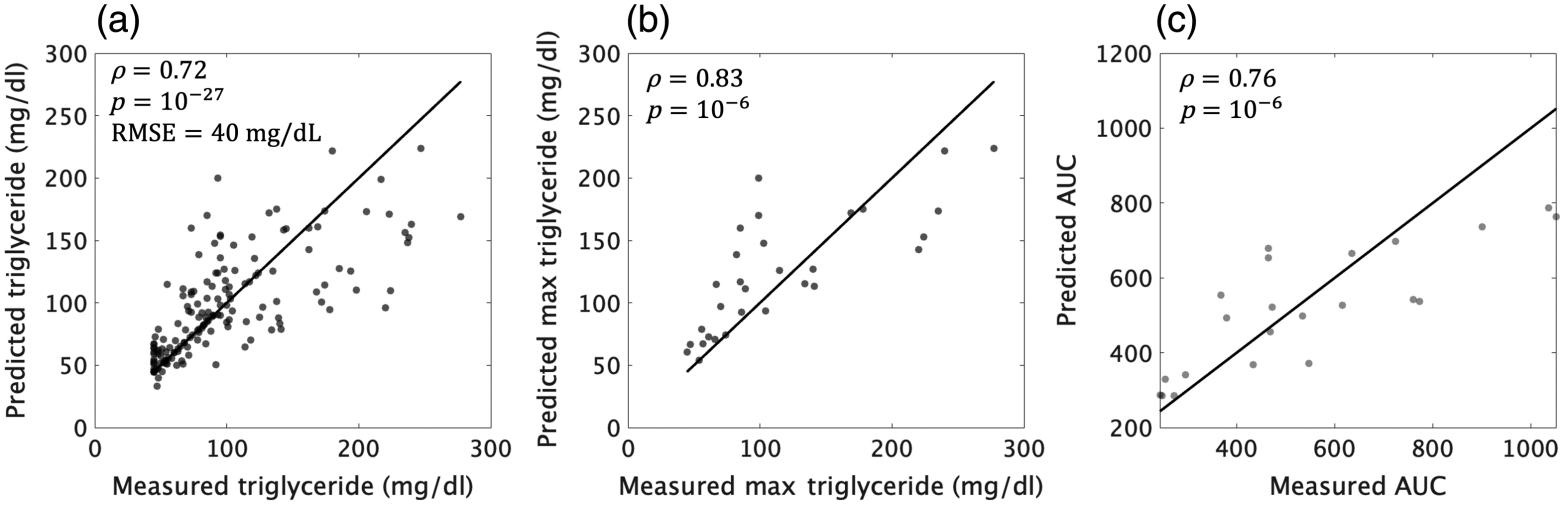
Predictive analysis of triglyceride concentrations utilizing optical metrics with the XGBoost model: (a) A plot of the predicted versus the actual triglyceride concentrations for all subjects across all timepoints. (b) A comparison of the predicted and measured area under the curve (AUC) for triglyceride concentration during the postprandial state. (c) The peak triglyceride concentrations as predicted by the model against the observed maximum values post-meal for each participant.

Figure 5(a) shows predicted versus known triglyceride concentrations in the postprandial state. The correlation coefficient between the predicted and measured values was found to be 0.72 (with a p-value of $10^{-27}$), and the root mean square error (RMSE) of 40  mg/dL. We further explored the ability to predict two key postprandial metrics: the area under the curve (AUC) and the peak triglyceride concentration during the longitudinal assessment. These comparisons are represented in Figs. 5(b) and 5(c), with Pearson correlation coefficients of 0.83 and 0.76, respectively. These results demonstrate the potential of optical measurements to predict important postprandial parameters in this subject population.

## 4 Discussion

In this work, we explored the application of SFDI for non-invasive monitoring of meal-induced changes in optical properties and hemodynamics measured on the dorsum of the hand. We found marked differences in the measured absorption coefficient value at 880 nm, ${\mathrm{StO}}_{2}$, ${\mathrm{HbO}}_{2}$ concentration, and ratios of $\frac{\mu_{a}(880\;\mathrm{nm})}{\mu_{a}(730\;\mathrm{nm})}$, $\frac{\mu_{a}(1100\;\mathrm{nm})}{\mu_{a}(730\;\mathrm{nm})}$ between high- and low-fat meals. All these metrics increased after the high-fat meal and decreased after the low-fat meal. The peak of these changes occurred at 3 to 4 h in the high-fat meal cohort, which largely corresponds to the peak in plasma triglyceride levels. SFDI metrics were predictive of triglyceride levels using a machine-learning model. Together, these results highlight that peripheral tissue optical properties and oxygenation are linked to meal type in the postprandial state and support the potential for non-invasive SFDI to be used in the setting of dynamic responses to nutrient intake.

Although limited, measures of peripheral hemodynamics in the postprandial state have been previously pursued.^11^^,^^12^^,^^19^^–^^21^ By far, the most common method is the so-called flow-mediated dilation procedure, in which the change in diameter of the brachial artery is measured using ultrasound following the release of an arterial occlusion by the upper arm cuff. Several studies have demonstrated an impairment of brachial artery dilation in the 6-h period following a high-fat meal, whereas other reports indicate a potentially meaningful correlation between this impairment and changes in circulating triglyceride levels.^19^ This impairment has been hypothesized to be caused by the production of superoxide and/or oxygen free radicals, causing an inhibition of endothelial production of nitric oxide, an established vasodilator.^19^ Other studies have shown an increase in blood flow to the digestive system, i.e., postprandial hyperemia, following a meal,^11^^,^^12^ which often occurs rapidly, 1 to 2 h into the postprandial state. Others have noted changes in systemic blood pressure after a meal, including a drop in systolic and diastolic blood pressure (i.e., postprandial hypotension) that peaks within the first 2 h after a meal.^20^^,^^21^

The hemodynamic measurements and temporal patterns observed here are distinct from prior observations in several important ways. First, flow-mediated dilation studies focus on large-diameter conduit arteries, typically in the arm, and studies of postprandial hyperemia have focused on large arteries superior mesenteric artery,^22^ whereas the changes measured in this study were in superficial tissue, which primarily comprises the microvasculature, including minor arterioles and venules. Second, whereas prior studies have measured blood flow, vessel diameter, or systemic hemodynamic measures such as blood pressure, we measured changes in tissue optical properties and functional metrics such as ${\mathrm{StO}}_{2}$ and ${\mathrm{HbO}}_{2}$ concentration. Most importantly, we report here that meal type leads to distinct patterns in peripheral tissue optical properties and hemodynamics that are distinct, although potentially linked in some cases, to the observations in these prior postprandial studies. For example, the decrease in ${\mathrm{HbO}}_{2}$ and ${\mathrm{StO}}_{2}$ in the first hour after a high-fat meal may be related to postprandial hyperemia, in which the shunting of blood occurs to digestive organs and away from the periphery.^11^^,^^12^ The subsequent increases in ${\mathrm{HbO}}_{2}$ and ${\mathrm{StO}}_{2}$ in the next several hours after a high-fat meal may be related to changes in vascular tone, tissue metabolic rate, systemic blood pressure, or others. Of note, these changes occurred in a temporal pattern that matched the trends of plasma triglycerides, suggesting a potential relationship between these parameters. It is also of note that systolic blood pressure increased in the high-fat cohort and decreased in the low-fat cohort, although statistical significance was only present at hour 2. Some prior studies have observed statistical increases in systolic blood pressure several hours after the ingestion of meals containing high levels of salt, although the amount of salt consumed in these studies was larger than in the current study (3 or 6 g of sodium versus <1.7  g of sodium for the high-fat meal in this study).^23^^,^^24^

One potential application of these observations made here is in the development of a non-invasive method to track postprandial responses. We took the first steps toward this goal by showing that triglyceride levels in the postprandial state could be predicted with relatively high accuracy (RMSE of 40  mg/dL) using a machine learning model involving 16 SFDI metrics. The information provided by the model may be sufficient for the identification of trends related to diet or specific meals on a day-to-day basis. Furthermore, the model was able to predict the peak triglyceride concentration and the AUC in the blood post-consumption with high agreement with the gold standard (Pearson correlation coefficient of 0.83 and 0.76, respectively). These metrics have been shown to signal abnormal lipid metabolism if found to be elevated.^25^^,^^26^

We notably did not find any significant difference in optical scattering metrics between the high-fat and low-fat meals at any time point in our study. In our prior work, we estimated a 2 to 4% change in blood $\mu_{s}^{\prime }$ at the three measurement wavelengths used here for healthy subjects after a high-fat meal due to alterations in size, number density, and refractive index of lipoprotein particles.^27^ However, these changes primarily occur in the blood, which constitutes only a small percentage of the overall tissue. As a result, the magnitude of scattering changes may be too small to be detected when measuring the optical properties of tissue using SFDI in healthy volunteers. That being said, patients suffering from dyslipidemias such as type II diabetes or hypertriglyceridemia can have much larger increases in plasma triglycerides (>10× compared with those observed here),^28^ which may be detectable non-invasively using techniques such as SFDI.

The selection of wavelengths for this study was strategically made to enable the measurement of hemoglobin levels by utilizing wavelengths positioned around the isosbestic point. In addition, we incorporated an SWIR wavelength of 1100 nm, chosen for its capability to achieve deeper tissue penetration due to the reduced absorption and scattering of light by tissue at this wavelength.^18^ This deeper penetration facilitates the examination of subcutaneous tissue and blood properties beneath the skin. We observed that the $\mu_{a}$ at 1100 nm, along with the composite metrics of $\mu_{a}$ at this wavelength, exhibited a strong correlation with postprandial triglyceride concentration.

There were several important limitations of this study. First, this study does not elucidate the underlying mechanisms responsible for the observed changes in SFDI-measured parameters. In the future, additional measurements of microvascular blood flow using techniques such as diffuse correlation spectroscopy (DCS) or speckle contrast optical spectroscopy (SCOS) may help further contextualize the observed changes in $\mu_{a}$, ${\mathrm{StO}}_{2}$, and ${\mathrm{HbO}}_{2}$ concentration, allowing changes in oxygen consumption from the oxygen supply to be disentangled.^29^ In addition, it was not possible to disentangle the effects of different consumed nutrients and lipid types between the meals. In addition to the higher calorie and fat content, the high-fat meals used in this study contained higher amounts of salt and sugar compared with the low-fat meals. This introduces other nutritional variables that could potentially influence cardiovascular metrics. There also may have been variability in the preparation of the breakfast items purchased from external vendors such as Dunkin Donuts and McDonalds, potentially affecting the consistency of the nutrient content across servings. Participants’ activities were not directly monitored between measurements, which may introduce variability in the study results. Finally, the demographic and health status of the participants were another limitation of the study, as all subjects were young and healthy.

Building on our findings, future research can now consider other populations in which postprandial changes are more likely to be present, including older individuals, patients with known dyslipidemias, and those with diabetes or prediabetes, all settings in which dynamic, postprandial changes in triglyceride concentrations are often more pronounced and could lead to more observable optical effects, including detectable changes in scattering properties.

In summary, the work presented here shows for the first time that SFDI can noninvasively track postprandial changes. Our findings indicate a robust relationship between several SFDI-derived metrics, including ${\mathrm{StO}}_{2}$ and ${\mathrm{HbO}}_{2}$ concentration, and blood triglyceride levels. These results have implications for diet monitoring and, in the future, may provide insight into vascular health for individuals with dyslipidemias and cardiovascular disease.

## Supplementary Material

10.1117/1.BIOS.1.2.025004.s01

## Data Availability

Data points contained in the figures of this manuscript have been uploaded as part of the manuscript package.

## References

[1] Bansal S. et al., “Fasting compared with nonfasting triglycerides and risk of cardiovascular events in women,” JAMA 298, 309–316 (2007). JAMAAP 0098-7484 10.1001/jama.298.3.309 17635891

[2] Daenen S. et al., “Peak-time determination of post-meal glucose excursions in insulin-treated diabetic patients,” Diabetes Metab. 36, 165–169 (2010). 10.1016/j.diabet.2009.12.002 20226708

[3] Keirns B. H. et al., “Fasting, non-fasting and postprandial triglycerides for screening cardiometabolic risk,” J. Nutr. Sci. 10, e75 (2021). JNSVA5 0301-4800 10.1017/jns.2021.73 34589207 PMC8453457

[4] Bergman M. et al., “Fasting, non-fasting and postprandial triglycerides for screening cardiometabolic risk,” Diabetes Res. Clin. Pract. 10, e75 (2020). DRCPE9 0168-8227 10.1017/jns.2021.73 PMC8453457 34589207

[5] Boquist S. et al., “Insulin and non-esterified fatty acid relations to alimentary lipaemia and plasma concentrations of postprandial triglyceride-rich lipoproteins in healthy middle-aged men,” Diabetologia 43, 185–193 (2000). DBTGAJ 0012-186X 10.1007/s001250050028 10753040

[6] Stanhope K. L. et al., “Consumption of fructose and high fructose corn syrup increase postprandial triglycerides, LDL-cholesterol, and apolipoprotein-B in young men and women,” J. Clin. Endocrinol. Metab. 96, E1596–E605 (2011). 10.1210/jc.2011-1251 21849529 PMC3200248

[7] Ras R. T. et al., “Flow-mediated dilation and cardiovascular risk prediction: a systematic review with meta-analysis,” Int. J. Cardiol. 168, 344–351 (2013). IJCDD5 0167-5273 10.1016/j.ijcard.2012.09.047 23041097

[r8] Korkmaz H. Onalan O., “Evaluation of endothelial dysfunction: flow-mediated dilation,” Endothelium 15, 157–163 (2008). ENDTE9 1062-3329 10.1080/10623320802228872 18663619

[r9] Vogel R. A. Corretti M. C. Plotnick G. D., “Effect of a single high-fat meal on endothelial function in healthy subjects,” Am. J. Cardiol. 79, 350–354 (1997). AJNCE4 0258-4425 10.1016/S0002-9149(96)00760-6 9036757

[10] Giannattasio C. et al., “Acute effect of high-fat meal on endothelial function in moderately dyslipidemic subjects,” Arterioscler. Thromb. Vasc. Biol. 25, 406–410 (2005). ATVBFA 1079-5642 10.1161/01.ATV.0000152231.93590.17 15576637

[11] Jeays A. D. et al., “A framework for the modeling of gut blood flow regulation and postprandial hyperaemia,” World J. Gastroenterol. 13, 1393–1398 (2007). 10.3748/wjg.v13.i9.1393 17457971 PMC4146924

[12] Gallavan R. H. Chou C. C., “Possible mechanisms for the initiation and maintenance of postprandial intestinal hyperemia,” Am. J. Physiol. 249, G301–G308 (1985). AJPHAP 0002-9513 10.1152/ajpgi.1985.249.3.G301 3898869

[13] Cuccia D. J. et al., “Quantitation and mapping of tissue optical properties using modulated imaging,” J. Biomed. Opt. 14, 024012 (2009). JBOPFO 1083-3668 10.1117/1.3088140 19405742 PMC2868524

[14] Pilvar A. et al., “Shortwave infrared spatial frequency domain imaging for non-invasive measurement of tissue and blood optical properties,” J. Biomed. Opt. 27, 066003 (2022). JBOPFO 1083-3668 10.1117/1.JBO.27.6.066003 35715883 PMC9204261

[15] Flock S. T. et al., “Optical properties of Intralipid: a phantom medium for light propagation studies,” Lasers Surg. Med. 12, 510–519 (1992). LSMEDI 0196-8092 10.1002/lsm.1900120510 1406004

[16] Allen T. J. et al., “Spectroscopic photoacoustic imaging of lipid-rich plaques in the human aorta in the 740 to 1400 nm wavelength range,” J. Biomed. Opt. 17, 061209 (2012). JBOPFO 1083-3668 10.1117/1.JBO.17.6.061209 22734739

[17] Segelstein D. J., The Complex Refractive Index of Water, University of Missouri, Kansas City (1981).

[18] Zhao Y. et al., “Shortwave-infrared meso-patterned imaging enables label-free mapping of tissue water and lipid content,” Nat. Commun. 11, 5355 (2020). NCAOBW 2041-1723 10.1038/s41467-020-19128-7 33097705 PMC7585425

[19] Plotnick G. D. Corretti M. C. Vogel R. A., “Effect of antioxidant vitamins on the transient impairment of endothelium-dependent brachial artery vasoactivity following a single high-fat meal,” JAMA 278(20), 1682–1686 (1997). JAMAAP 0098-7484 10.1001/jama.1997.03550200058032 9388088

[r20] Luciano G. L. Brennan M. J. Rothberg M. B., “Postprandial hypotension,” Am. J. Med. 123, 281.e1–281.e6 (2010). 10.1016/j.amjmed.2009.06.026 20193838

[21] Ahuja K. D. K. Robertson I. K. Ball M. J., “Acute effects of food on postprandial blood pressure and measures of arterial stiffness in healthy humans,” Am. J. Clin. Nutr. 90, 298–303 (2009). 10.3945/ajcn.2009.27771 19535430

[22] Moneta G. L. et al., “Duplex ultrasound measurement of postprandial intestinal blood flow: effect of meal composition,” Gastroenterology 95, 1294–1301 (1988). GASTAB 0016-5085 10.1016/0016-5085(88)90364-2 3049214

[23] Kanbay M. et al., “Acute effects of salt on blood pressure are mediated by serum osmolality,” J. Clin. Hypertens. 20, 1447–1454 (2018). JCHYEM 0748-450X 10.1111/jch.13374 PMC8030773 30232829

[24] Suckling R. J. et al., “Dietary salt influences postprandial plasma sodium concentration and systolic blood pressure,” Kidney Int. 81, 407–411 (2012). 10.1038/ki.2011.369 22048126

[25] Wilson S. M. et al., “Determinants of the postprandial triglyceride response to a high-fat meal in healthy overweight and obese adults,” Lipids Health Dis. 20, 107 (2021). 10.1186/s12944-021-01543-4 34544430 PMC8451105

[26] Lee D. P. S. et al., “The influence of different foods and food ingredients on acute postprandial triglyceride response: a systematic literature review and meta-analysis of randomized controlled trials,” Adv. Nutr. 11, 1529–1543 (2020). 10.1093/advances/nmaa074 32609800 PMC7666897

[27] Pilvar A. et al., “Feasibility of postprandial optical scattering of lipoproteins in blood as an optical marker of cardiovascular disease risk: modeling and experimental validation,” J. Biomed. Opt. 28, 065002 (2023). JBOPFO 1083-3668 10.1117/1.JBO.28.6.065002 37305780 PMC10249051

[28] Nordestgaard B. G. et al., “Fasting is not routinely required for determination of a lipid profile: clinical and laboratory implications including flagging at desirable concentration cut-points-a joint consensus statement from the European Atherosclerosis Society and European Feder,” Eur. Heart J. England 37, 1944–1958 (2016). 10.1093/eurheartj/ehw152 PMC4929379 27122601

[29] Carp S. A. Robinson M. B. Franceschini M. A., “Diffuse correlation spectroscopy: current status and future outlook,” Neurophotonics 10, 013509 (2023). 10.1117/1.NPh.10.1.013509 36704720 PMC9871606

