# Supplementary material for: Enhanced peripheral tissue oxygenation and hemoglobin concentration after a high-fat meal measured with spatial frequency domain imaging
Source: Biophotonics Discov. 2024 Sep 12;1(2):025004. doi: 10.1117/1.BIOS.1.2.025004 (PMC13098637; doi:10.1117/1.BIOS.1.2.025004)
Supplement: Supplementary file 1 [file BIOS_001_025004_SD001.docx]

**Supplementary Material**

**Table 1. Nutrition facts for the meal provided for subjects for low-fat and high-fat meal study**

|  | **High fat meal** | | | | **Low fat meal** |
| --- | --- | --- | --- | --- | --- |
|  | Bacon egg and cheese on croissant | Sausage, egg and cheese on bagel | McDonalds big breakfast | Ensure drink | Oatmeal |
| **Fat** | 36 g | 34 g | 52 g | 11 g | 2 g |
| **Sodium** | 820 mg | 1500 mg | 1477 mg | 210 mg | 240 mg |
| **Sugar** | 6 g | 8 g | 2.5 g | 22 g | 4 g |
| **Total Carb** | 41 g | 68 g | 47 g | 49 g | 24 g |
| **Calories** | 560 KCal | 680 KCal | 766 KCal | 350 KCal | 120 KCal |

**Segmentation process:** Larger vessel and microvascular regions were segmented by enhancing the absorption coefficient (μ_a_​) map at 880 nm using the fibermetric function in MATLAB. This function is designed to detect tubular structures in an image by evaluating the eigenvalues of the Hessian matrix. We enhanced bright structures with a specified size (50) and structure sensitivity (8), settings chosen empirically for optimal fiber-like structure detection. After enhancing the image, we normalized the output and created a threshold-based mask that correspond to the top 60% of the normalized values. We then generated a binary mask to separate pixels into vascular or microvascular regions. Pixels above this threshold were identified as part of the vascular regions, while the remainder were classified as microvascular. We used the number of pixels identified as vasculature to compare the size of the vasculature over time in the postprandial state. No significant change or difference between low-fat and high-fat meal was observed in vasculature size.

**FWHM analysis:** To determine whether meal consumption influenced vessel diameters, we examined a line profile across a large superficial vessel on the hand using the $\mu_{a}$​ map at 880 nm. A Gaussian curve was fitted to this profile, and the FWHM was calculated from the fit parameters. We subsequently assessed the average variation in FWHM among all subjects following the consumption of low-fat and high-fat meals over the course of the study. Our analysis revealed no significant changes or differences in the FWHM measurements post-meal.


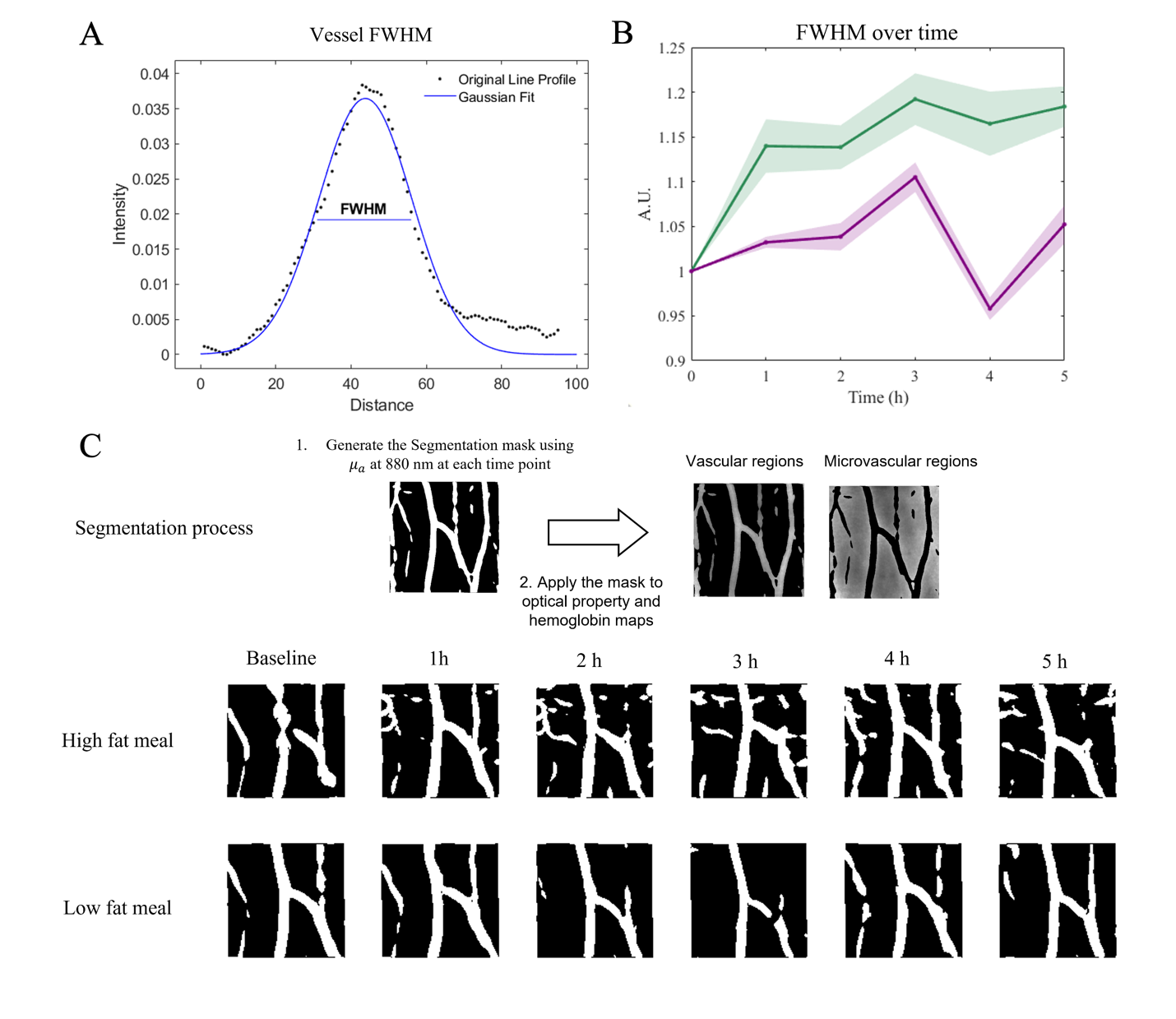


Fig 1. A) line profile over a large superficial vessel and the Gaussian fit used to calculate FWHM. B) FWHM changes after low-fat and high-fat meal over one superficial vessel for all 15 subjects. The shaded area shows the standard error. C) The segmentation process and the segmented vessel after high-fat and low-fat meal for one nominated subject.
